# Genome-wide analysis of mitochondrial DNA copy number reveals loci implicated in nucleotide metabolism, platelet activation, and megakaryocyte proliferation

**DOI:** 10.1007/s00439-021-02394-w

**Published:** 2021-12-02

**Authors:** R. J. Longchamps, S. Y. Yang, C. A. Castellani, W. Shi, J. Lane, M. L. Grove, T. M. Bartz, C. Sarnowski, C. Liu, K. Burrows, A. L. Guyatt, T. R. Gaunt, T. Kacprowski, J. Yang, P. L. De Jager, L. Yu, A. Bergman, R. Xia, M. Fornage, M. F. Feitosa, M. K. Wojczynski, A. T. Kraja, M. A. Province, N. Amin, F. Rivadeneira, H. Tiemeier, A. G. Uitterlinden, L. Broer, J. B. J. Van Meurs, C. M. Van Duijn, L. M. Raffield, L. Lange, S. S. Rich, R. N. Lemaitre, M. O. Goodarzi, C. M. Sitlani, A. C. Y. Mak, D. A. Bennett, S. Rodriguez, J. M. Murabito, K. L. Lunetta, N. Sotoodehnia, G. Atzmon, K. Ye, N. Barzilai, J. A. Brody, B. M. Psaty, K. D. Taylor, J. I. Rotter, E. Boerwinkle, N. Pankratz, D. E. Arking

**Affiliations:** 1 Department of Genetic Medicine, McKusick-Nathans Institute, Johns Hopkins University School of Medicine, Baltimore, MD USA; 2 Department of Pathology and Laboratory Medicine, Western University, London, ON Canada; 3 Department of Laboratory Medicine and Pathology, University of Minnesota Medical School, Minneapolis, MN USA; 4 Department of Epidemiology, Human Genetics, and Environmental Sciences, School of Public Health, Human Genetics Center, The University of Texas Health Science Center at Houston, Houston, TX USA; 5 Cardiovascular Health Research Unit, Departments of Medicine and Biostatistics, University of Washington, Seattle, WA USA; 6 Department of Biostatistics, Boston University School of Public Health, Boston, MA USA; 7 MRC Integrative Epidemiology Unit at the University of Bristol, University of Bristol, Oakfield House, Oakfield Grove, Bristol, UK; 8 Population Health Sciences, Bristol Medical School, University of Bristol, Oakfield House, Oakfield Grove, Bristol, UK; 9 Department of Health Sciences, University of Leicester, University Road, Leicester, UK; 10 Department of Functional Genomics, Interfaculty Institute for Genetics and Functional Genomics, University of Greifswald, Greifswald, Germany; 11 Rush Alzheimer’s Disease Center and Department of Neurological Sciences, Rush University Medical Center, Chicago, IL USA; 12 Center for Translational and Systems Neuroimmunology, Department of Neurology, Columbia University Medical Center, New York, NY USA; 13 Program in Medical and Population Genetics, Broad Institute, Cambridge, MA USA; 14 Department of Systems and Computational Biology, Albert Einstein College of Medicine, Bronx, NY USA; 15 Institute of Molecular Medicine, The University of Texas Health Science Center at Houston, Houston, TX USA; 16 Human Genetics Center, The University of Texas Health Science Center at Houston, Houston, USA; 17 Division of Statistical Genomics, Department of Genetics, Washington University School of Medicine, St. Louis, USA; 18 Department of Epidemiology, Erasmus Medical Center, Rotterdam, The Netherlands; 19 Department of Internal Medicine, Erasmus Medical Center, Rotterdam, The Netherlands; 20 Department of Social and Behavioral Science, Harvard T.H. School of Public Health, Boston, USA; 21 Department of Genetics, University of North Carolina at Chapel Hill, Chapel Hill, NC USA; 22 Department of Medicine, University of Colorado Denver, Anschutz Medical Campus, Aurora, CO USA; 23 Center for Public Health Genomics, University of Virginia, Charlottesville, VA USA; 24 Cardiovascular Health Research Unit, Department of Medicine, University of Washington, Seattle, WA USA; 25 Division of Endocrinology, Diabetes and Metabolism, Cedars-Sinai Medical Center, Los Angeles, CA USA; 26 Cardiovascular Research Institute and Institute for Human Genetics, University of California, San Francisco, CA USA; 27 Boston University School of Medicine, Boston University, Boston, MA USA; 28 Cardiovascular Health Research Unit, Division of Cardiology, University of Washington, Seattle, WA USA; 29 Department of Natural Science, University of Haifa, Haifa, Israel; 30 Department of Epidemiology and Population Health, Albert Einstein College of Medicine, Bronx, NY 10461 USA; 31 Departments of Medicine and Genetics, Albert Einstein College of Medicine, Bronx, NY 10461 USA; 32 Cardiovascular Health Research Unit, Departments of Epidemiology, Medicine and Health Services, University of Washington, Seattle, WA USA; 33 The Institute for Translational Genomics and Population Sciences, Department of Pediatrics, The Lundquist Institute for Biomedical Innovation at Harbor-UCLA Medical Center, Torrance, CA USA; 34 Baylor College of Medicine, Human Genome Sequencing Center, Houston, TX USA; 35 Data Science in Biomedicine, Peter L. Reichertz Institute for Medical Informatics, TU Braunschweig and Hannover Medical School, Brunswick, Germany

## Abstract

Mitochondrial DNA copy number (mtDNA-CN) measured from blood specimens is a minimally invasive marker of mitochondrial function that exhibits both inter-individual and intercellular variation. To identify genes involved in regulating mitochondrial function, we performed a genome-wide association study (GWAS) in 465,809 White individuals from the Cohorts for Heart and Aging Research in Genomic Epidemiology (CHARGE) consortium and the UK Biobank (UKB). We identified 133 SNPs with statistically significant, independent effects associated with mtDNA-CN across 100 loci. A combination of fine-mapping, variant annotation, and co-localization analyses was used to prioritize genes within each of the 133 independent sites. Putative causal genes were enriched for known mitochondrial DNA depletion syndromes (*p* = 3.09 × 10^–15^) and the gene ontology (GO) terms for mtDNA metabolism (*p* = 1.43 × 10^–8^) and mtDNA replication (*p* = 1.2 × 10^–7^). A clustering approach leveraged pleiotropy between mtDNA-CN associated SNPs and 41 mtDNA-CN associated phenotypes to identify functional domains, revealing three distinct groups, including platelet activation, megakaryocyte proliferation, and mtDNA metabolism. Finally, using mitochondrial SNPs, we establish causal relationships between mitochondrial function and a variety of blood cell-related traits, kidney function, liver function and overall (*p* = 0.044) and non-cancer mortality (*p* = 6.56 × 10^–4^).

**Supplementary Information:**

The online version contains supplementary material available at 10.1007/s00439-021-02394-w.

## Introduction

Mitochondria are the cellular organelles primarily responsible for producing the chemical energy required for metabolism, as well as signaling the apoptotic process, maintaining homeostasis, and synthesizing several macromolecules such as lipids, heme and iron-sulfur clusters (Wallace 1992; Vakifahmetoglu-Norberg et al. 2017). Mitochondria possess their own genome (mtDNA); a circular, intron-free, double-stranded, haploid, ~ 16.6 kb maternally inherited molecule encoding 37 genes vital for proper mitochondrial function. Due to the integral role of mitochondria in cellular metabolism, mitochondrial dysfunction is known to play a critical role in the underlying etiology of several aging-related diseases (Dai et al. 2012; Cui et al. 2012; Herst et al. 2017).

Unlike the nuclear genome, a large amount of variation exists in the number of copies of mtDNA present within cells, tissues, and individuals. The relative copy number of mtDNA (mtDNA-CN) has been shown to be positively correlated with oxidative stress (Liu et al. 2003), energy reserves, and mitochondrial membrane potential (Guha and Avadhani 2013). As a minimally invasive proxy measure of mitochondrial dysfunction (Malik and Czajka 2013), decreased mtDNA-CN measured in blood has been previously associated with aging-related disease states including frailty (Ashar et al. 2015), cardiovascular disease (Ashar et al. 2017; Hong et al. 2020; Zhao et al. 2020), chronic kidney disease (Tin et al. 2016), neurodegeneration (Pyle et al. 2016; Wei et al. 2017), and cancer (Reznik et al. 2016).

Although mtDNA-CN measured from whole blood presents itself as an easily accessible and minimally invasive biomarker, cell-type composition has been shown to be an important confounder, complicating analyses (Hurtado-Roca et al. 2016; Knez et al. 2016). For example, while platelets generally have fewer mtDNA molecules than leukocytes, the lack of a platelet nuclear genome drastically skews mtDNA-CN estimates. As a result, not only is controlling for cell composition extremely vital for accurate mtDNA-CN estimation, interpreting the results in relation to the impact of cell composition becomes a necessity (Knez et al. 2016; Kumar et al. 2018; Urata et al. 2008).

Though the comprehensive mechanism through which mtDNA-CN is modulated is largely unknown (Clay Montier et al. 2009; Tang et al. 2000), twin studies have estimated a broad-sense heritability of ~ 0.65, consistent with moderate genetic control (Xing et al. 2008). Several nuclear genes have been shown to directly modulate mtDNA-CN, specifically those within the mtDNA replication machinery such as the mitochondrial polymerase, *POLG* and *POLG2* (Carling et al. 2011; Harvey et al. 2011), as well as the mitochondrial DNA helicase, *TWNK*, and the mitochondrial single-stranded binding protein, *mtSSB* (Copeland 2014). It is important to note that mtDNA-CN and mitochondrial transcription are intertwined, as many mitochondrial factors are involved in both mitochondrial replication and transcription (Clayton 2000). Furthermore, nuclear genes which maintain proper mitochondrial nucleotide supply including *DGUOK* and *TK2* have also been shown to regulate mtDNA-CN (Mandel et al. 2001; Wang et al. 2005; Rusecka et al. 2018). To further elucidate the genetic control over mtDNA-CN, several genome-wide association studies (GWAS) of mtDNA-CN have been published (Cai et al. 2015; Workalemahu et al. 2017; Guyatt et al. 2019; Hägg et al. 2020), including a study that was published while the current manuscript was in preparation, analyzing ~ 300,000 participants from the UK Biobank (UKB), and identifying 50 independent loci (Hägg et al. 2020).

SNPs located on the mtDNA genome presumably affect different biological pathways solely through mitochondrial function. mtDNA SNPs have previously been shown to be associated with oxidative consumption and gene expression (Cohen et al. 2016; Gómez-Durán et al. 2010). Additionally, mtDNA-SNPs are known to associate with altered risks of developing many diseases, and can modulate mitochondrial protein translation (Marom et al. 2017; Cai et al. 2021). To dissect whether mitochondrial processes are causal for diseases associated with mtDNA-CN, we utilized mtDNA-SNPs as a proxy for mitochondrial function.

In the present study, we report mtDNA-CN GWAS results from 465,809 individuals across the Cohorts for Heart and Aging Research in Genomic Epidemiology (CHARGE) consortium (Psaty et al. 2009) and the UK Biobank (UKB) (Bycroft et al. 2018). Using multiple gene prioritization and functional annotation methods, we assign genes to loci that reached genome-wide significance. We perform a PHEWAS and group our genome-wide significant SNPs into three clusters that represent distinct functional domains related to mtDNA-CN. Finally, we leverage mitochondrial SNPs to establish causality between mitochondrial function and mtDNA-CN associated traits.

## Subjects and methods

### Study populations

470,579 individuals participated in this GWAS, 465,809 of whom self-identified as White. Participants were derived from 7 population-based cohorts representing the Cohorts for Heart and Aging Research in Genetic Epidemiology (CHARGE) consortium (Avon Longitudinal Study of Parents and Children [ALSPAC], Atherosclerosis Risk in Communities [ARIC], Cardiovascular Health Study [CHS], Multi-Ethnic Study of Atherosclerosis [MESA], Religious Orders Study and Memory and Aging Project [ROSMAP], Study of Health in Pomerania [SHIP]) and from the UK Biobank (UKB) (Supplemental Table 1). Detailed descriptions of each participating cohort, their quality control practices, study level analyses, and ethic statements are available in the Supplemental Methods. All study participants provided written informed consent and all centers obtained approval from their institutional review boards.

### Methods for mitochondrial DNA copy number estimation (CHARGE cohorts)

#### qPCR

mtDNA-CN was determined using a quantitative PCR assay as previously described (Guyatt et al. 2019; Longchamps et al. 2020). Briefly, the cycle threshold (Ct) value of a nuclear-specific and mitochondrial-specific probe were measured in triplicate for each sample. In CHS, a multiplex assay using the mitochondrial *ND1* probe and nuclear *RPPH1* probe was used, whereas ALSPAC used a mitochondrial probe targeting the D-Loop and a nuclear probe targeting *B2M*. In CHS, we observed plate effects, as well as a linear increase in Δ*C*t due to the pipetting order of each replicate. These effects were corrected in the analysis using linear mixed model regression, with pipetting order included as a fixed effect and plate as a random effect to create a raw measure of mtDNA-CN prior to correcting for mtDNA-CN associated covariates such as age and sex. In ALSPAC, run-to-run variability was controlled using three calibrator samples added to every plate, to allow for adjustment by a per-plate calibration factor (Guyatt et al. 2019). CHS DNA was extracted by salt precipitation following proteinase K digestion of the buffy coat from whole blood, while ALSPAC DNA was extracted using a phenol–chloroform method (Ashar et al. 2017; Guyatt et al. 2019).

#### Microarray

Microarray probe intensities were used to estimate mtDNA-CN using the Genvisis software package (MitoPipeline 2021) as previously described (Ashar et al. 2017; Longchamps et al. 2020). Briefly, Genvisis uses the median mitochondrial probe intensity across all homozygous mitochondrial SNPs as an initial estimate of mtDNA-CN. Technical artifacts such as DNA input quality, DNA input quantity, and hybridization efficiency were captured through either surrogate variable (SV) or principal component (PC) analyses. SVs or PCs were adjusted for through stepwise linear regression by adding successive components until each successive surrogate variable or principal component no longer significantly improved the model.

#### Whole-genome sequencing (ARIC)

Whole-genome sequencing read counts were used to estimate mtDNA-CN as previously described (Longchamps et al. 2020). Briefly, the total number of reads in a sample were web scraped from the NCBI sequence read archive. Mitochondrial reads were downloaded directly from dbGaP through Samtools (1.3.1). There was no overlap between ARIC microarray and ARIC whole-genome sequencing samples. A ratio of mitochondrial reads to total aligned reads was used as a raw measure of mtDNA-CN.

#### Adjusting for covariates

Each method described above represents a raw measure of mtDNA-CN, adjusted for technical artifacts; however, several potential confounding variables (e.g., age, sex, blood cell composition) have been identified previously (Knez et al. 2016). Raw mtDNA-CN values were adjusted for white blood cell count via linear regression in ARIC, SHIP and CHS (which also adjusted for platelet count), depending on available data. For all studies, standardized residuals (mean = 0, standard deviation = 1) of mtDNA-CN were used after adjusting for covariates (Supplemental Table 1).

### Estimation of mitochondrial DNA copy number (UKB)

Due to the availability of more detailed cell count data, as well as a different underlying biochemistry for the Affymetrix Axiom array compared to the genotyping arrays used in the CHARGE cohorts, mtDNA-CN in the UKB was estimated differently (Supplemental Methods). Briefly, mtDNA-CN estimates derived from whole-exome sequencing data, available on ~ 50,000 individuals, were generated first using customized Perl scripts to aggregate the number of mapped sequencing reads and correct for covariates through both linear and spline regression models. Concurrently, mitochondrial probe intensities from the Affymetrix Axiom arrays, available on the full ~ 500,000 UKB cohort, were adjusted for technical artifacts through principal components generated from nuclear probe intensities. Probe intensities were then regressed onto the whole-exome sequencing mtDNA-CN metric, and beta estimates from that regression were used to estimate mtDNA-CN in the full UKB cohort. Finally, we used a tenfold cross-validation method to select the cell counts to include in the final model (Supplemental Table 2). The final UKB mtDNA-CN metric is the standardized residuals (mean = 0, standard deviation = 1) from a linear model adjusting for covariates (age, sex, cell counts) as described in the Supplemental Methods.

### Genome-wide association study

For each individual cohort, regression analysis was performed with residualized mtDNA-CN as the dependent variable adjusting for age, sex, and cohort-specific covariates (e.g., principal components, DNA collection site, family structure, cell composition). Cohorts with multiple mtDNA-CN estimation platforms were stratified into separate analyses. Ancestry-stratified meta-analyses were performed using Metasoft software using the Han and Eskin random-effects model to control for unobserved heterogeneity due to differences in mtDNA-CN estimation method (Han and Eskin 2011). Effect size estimates for SNPs were calculated using a random-effect meta-analysis from cohort summary statistics, as the Han and Eskin model relaxes the assumption under the null hypothesis without modifying the effect size estimates that occur under the alternative hypothesis (Han and Eskin 2011). In total, three complementary analyses were performed in self-identified White individuals, (1) a meta-analysis using all available studies, (2) a meta-analysis of studies with available data for cell count adjustments, and (3) an analysis of UKB-only data. The UKB GWAS was performed on all self-identified white individuals, excluding individuals who were cell count outliers (See Supplemental Methods). Relatedness was accounted for using a kinship matrix. As the vast majority of samples are derived from the UKB study, and given the difficulty in interpreting effect size estimates from a random-effects model, further downstream analyses were all performed using effect size estimates from UKB-only data. We additionally performed X chromosome analyses using only UKB data. X-chromosome analyses were stratified by sex (males = 194,151, females = 216,989), and summary statistics were meta-analyzed using METAL (Willer et al. 2010) to obtain the final effect estimates (Supplemental Fig. 1).

### SNP heritability estimation

SNP heritability estimates were retrieved from BOLT-LMM (Loh et al. 2015). To verify this metric, we used SumHer (Speed 2019) to calculate an independent heritability metric using summary statistics. The heritability model used in this analysis was the BLD-LDAK model. The tagging file used is the pre-computed UK Biobank GBR version for the corresponding heritability model. The summary statistics were filtered so that only single-character reference and alternate alleles are allowed. Chr:BP combination duplicates were removed except for the first appearance. SNP heritability was then calculated and extracted from output files.

### Identification of independent GWAS loci

To identify the initial genome-wide significant (lead) SNPs in each locus, the most significant SNP that passed genome-wide significance (*p* < 5 × 10^–8^) within a 1 Mb window was selected. To avoid Type I error, SNPs were only retained for further analyses if there were either (a) at least two genome-wide significant SNPs in the 1 Mb window or (b) if the lead SNP was directly genotyped. Conditional analyses were performed in UKB, where the lead SNPs from the original GWAS were used as additional covariates to identify additional independent associations.

### Comparisons with Hägg et al. 2020

To compare results with Hägg et al. (2020), summary statistics were obtained from their Supplementary Table 4. Loci were identified as shared between the two GWAS if two lead SNPs were fewer than 500,000 base pairs apart from one another.

### Fine-mapping

The susieR package was used to identify all potential causal variants for each independent locus associated with mtDNA CN (Wang et al. 2020). UKB imputed genotype data for unrelated White subjects were used and variants were extracted using a 500 kb window around the lead SNP for each locus with minor allele frequency (MAF) > 0.001. 95% credible sets (CS) of SNPs, containing a potential causal variant within a locus, were generated. The minimum absolute correlation within each CS is 0.5 and the scaled prior variance is 0.01. When the CS did not include the lead SNP (six out of 96 loci) identified from the GWAS, some of the parameters were slightly relaxed [minimum absolute correlation is 0.2, estimate prior variance is TRUE]. The SNP with the highest posterior inclusion probability (PIP) within each CS was also identified (Supplemental Table 3). With a few exceptions, final lead SNPs were selected by prioritizing initially identified SNPs unless the SNP with the highest PIP had a PIP greater than 0.2 and was 1.75 times larger than the SNP with the second highest PIP.

### Functional annotation and gene prioritization

#### Functional annotation

ANNOVAR was used for functional annotation of variants identified in the fine-mapping step (Wang et al. 2010). First, variants were converted to an ANNOVAR-ready format using the dbSNP version 150 database (Sherry et al. 1999). Then, variants were annotated with ANNOVAR using the RefSeq Gene database (O’Leary et al. 2016). The annotation for each variant includes the associated gene and region (e.g., exonic, intronic, intergenic). For intergenic variants, ANNOVAR provides flanking genes and the distance to each gene. For exonic variants, annotations also include likely functional consequences (e.g., synonymous/nonsynonymous, insertion/deletion), the gene affected by the variant, and the amino acid sequence change (Supplemental Table 4).

#### Co-localization analyses

Co-localization analyses were performed using the approximate Bayes factor method in the R package *coloc* (Giambartolomei et al. 2014). Briefly, *coloc* utilizes eQTL data and GWAS summary statistics to evaluate the probability that gene expression and GWAS data share a single causal SNP (colocalize). *Coloc* returns multiple posterior probabilities; H0 (no causal variant), H1 (causal variant for gene expression only), H2 (causal variant for mtDNA-CN only), H3 (two distinct causal variants), and H4 (shared causal variant for gene expression and mtDNA-CN). In the event of high H4, we designate the gene as causal for the GWAS phenotype of interest (mtDNA-CN). eQTL summary statistics were obtained from the eQTLGen database (Võsa et al. 2018). Genes with significant associations with lead SNPs were tested for co-localization using variants within a 500 kb window of the sentinel SNP. Occasionally, some of the eQTLGen *p* values for certain SNPs were identical due to R’s (ver 4.0.3) limitation in handling small numbers. To account for this, if the absolute value for a SNP’s *z*-score association with a gene was greater than 37.02, *z*-scores were rescaled so that the largest *z*-score would result in a *p* value of 5 × 10^–300^. Additionally, a few clearly co-localized genes did not result in high H4 PPs due to the strong effect for each phenotype of a single SNP (Supplemental Fig. 2), possibly due to differences in linkage disequilibrium (LD) between the UKB and eQTLGen populations. To account for this, we summed mtDNA-CN GWAS *p* values and eQTLGen *p* values for each SNP and removed the SNP with the lowest combined *p* value. Co-localization analyses were then repeated without the lowest SNP. Genes with H4 greater than 50% were classified as genes with significant evidence of co-localization. The fifty percent cut-off was chosen based on visual inspection of plots. Ultimately, functional studies are necessary to prove true causality between candidate genes and mtDNA-CN.

#### DEPICT

Gene prioritization was performed with Depict, an integrative tool that incorporates gene co-regulation and GWAS data to identify the most likely causal gene at a given locus (Pers et al. 2015). Across GWAS SNPs which overlapped with the DEPICT database, we identified SNPs representing 119 independent loci with LD pruning defined as *p* < 5 × 10^–8^, *r*^2^ < 0.05 and > 500 kb from other locus boundaries. Only genes with a nominal *p* value of less than 0.05 were considered for downstream prioritization.

#### Gene assignment

To prioritize genes for each identified locus, we utilized functional annotations, eQTL co-localization analyses, and DEPICT gene prioritization results (Supplemental Fig. 3). First, genes with missense variants within susieR fine-mapped credible sets were assigned to loci. If loci co-localized with a gene’s expression with a posterior probability (PP) of greater than 0.50 and there were no other co-localized genes with a PP within 5%, the gene with the highest posterior probability was assigned. If there was still no assigned gene, the most significant DEPICT gene was assigned. If there was no co-localization or DEPICT evidence, the nearest gene was assigned.

### Gene set enrichment analyses

Using the finalized gene list from the prioritization pipeline, GO and KEGG pathway enrichment analyses were performed using the “goana” and “kegga” functions from the R package *limma* (Smyth et al. 2021), treating all known genes as the background universe (Young et al. 2010). Only one gene per locus was used for “goana” and “kegga” gene set enrichment analysis, prioritizing genes assigned to primary independent hits. If there were multiple assigned genes, one gene was randomly selected to avoid biasing results through loci with multiple functionally related genes. To identify an appropriate *p* value cutoff, 100 genes were randomly selected from the genome and run through the same enrichment analysis. This permutation was repeated 1000 times to generate a null distribution of the smallest *p* values from each permutation. For cluster-specific gene set enrichment analyses, permutation testing used the same number of random genes as the number of genes in each cluster. To ensure the robustness of results, gene set enrichment analysis was repeated 50 times with random selection of genes at loci with multiple assigned genes. GO and KEGG terms that passed permutation cutoffs at least 40/50 times were retained.

### Gene-based association test

We used metaXcan, which employs gene expression prediction models to evaluate associations between phenotypes and gene expression (Barbeira et al. 2018). We obtained pre-calculated expression prediction models and SNP covariance matrices, computed using whole blood from European ancestry individuals in version 7 of the Genotype-Tissue expression (GTEx) database (Barbeira et al. 2019). Using prediction performance *p* < 0.05, a total of 6285 genes were predicted. Of these genes, 74 passed Bonferroni correction of *p* < 7.95 × 10^–6^. Gene set enrichment analyses were performed on Bonferroni-significant genes as previously described. REVIGO (Supek et al. 2011) was used on the “medium” setting (allowed similarity = 0.7) to visualize significantly enriched GO terms.

We used a one-sided Fisher’s exact test to test for enrichment of genes that have been previously identified as causal for mtDNA depletion syndromes (Stiles et al. 2016; Kornblum et al. 2013; El-Hattab and Scaglia 2013).

### PHEWAS-based SNP clustering

#### mtDNA-CN phenome-wide association study (PHEWAS)

We used the PHEnome Scan ANalysis Tool (PHESANT) (Millard et al. 2018) to identify mtDNA-CN associated quantitative traits in the UKB. Briefly, we tested for the association of mtDNA-CN with 869 quantitative traits (Supplemental Table 5), limiting analyses to 365,781 White, unrelated individuals (used.in.pca.calculation = 1). As extreme cell count measurements could indicate individuals with active infections or cancers, they were excluded from analysis (see Supplemental Methods). Analyses were adjusted for age, sex, and assessment center.

#### SNP-phenotype associations

SNP genotypes were regressed on mtDNA-associated quantitative phenotypic traits using linear regression, adjusted for sex, age with a natural spline (d*f* = 2), assessment center, genotyping array, and 40 genotyping principal components (provided as part of the UKB data download).

#### SNP clustering

To identify distinct clusters of mtDNA-CN GWS SNPs based on phenotypic associations, beta estimates from the SNP-phenotype associations were first divided by the beta estimate of the mtDNA-CN SNP-mtDNA-CN association, so that all SNP-phenotype associations are relative to the mtDNA-CN increasing allele and scaled to the effect of the SNP on mtDNA-CN. The adjusted beta estimates were subjected to a dimensionality reduction method, Uniform Manifold and Approximation Projection (UMAP), as implemented in the R package *umap* (Konopka 2020) (random_state = 123, n_neighbors = 10, min_dist = 0.001, n_components = 2, n_epochs = 200). SNPs were assigned to clusters using Density-Based Clustering of Applications with Noise (DBSCAN) as implemented in the R package *dbscan* (Hahsler et al. 2019) (minPts = 10). Robustness of cluster assignment was established by varying n_neighors, min_dist, and random_state parameters. Clusters represent groups of SNPs with similar phenotypic associations.

#### Phenotype enrichment and permutation testing

To test for enrichment of *individual* phenotypes within clusters, we compared the median mtDNA-CN scaled phenotype beta estimates within the cluster to the median beta estimates for all SNPs not in the cluster, with significance determined using 20,000 permutations in which cluster assignment was permuted. For multi-test correction *across all* phenotypes, we performed 300 permutations of the initial cluster assignment, followed by the comparison of median beta estimates as described above. We retained only the most significant result from across all phenotypes and clusters from each of the 300 permutations, and then selected the 15th most significant value as the study-wide threshold for multi-test corrected significance of *p* < 0.05.

### mtDNA variant association analyses

#### Mitochondrial variant phasing and imputation

Shapeit4 and Impute5 were used for UK Biobank mtDNA genotype phasing and imputation (Delaneau et al. 2019; Rubinacci et al. 2020). Phasing and imputation were performed separately for each genotyping array (UKBB, UKBL), and restricted to self-identified White individuals. The reference panel used for imputation analysis was the 1000 Genomes Project phase 3 mtDNA variants (Auton et al. 2015). UK Biobank genotypes were coded to match the reference panel allele. All genotype files, including the reference panel, were phased using Shapeit4 to fill in any missing genotypes using the phasing iteration sequence “10b,1p,1b,1p,1b,1p,1b,1p,10 m”, where b is burn-in iteration, p is pruning iteration, and m is main iteration. The –sequencing option was also used due to the presence of multiple mtDNA variants in a very small region, analogous to sequencing data.

Phased UK Biobank genotypes were then imputed with the reference panel using Impute5 with the following parameters: –pbwt-depth 8; –pbwt-cm 0.005; –no-threshold. All imputed variants were functionally annotated using MSeqDR mvTools (Shen et al. 2018).

#### mtSNP association tests

Linear regressions stratified by genotyping array (UKBB, UKBL) were performed for each mtDNA SNP on the 41 traits and mtDNA-CN, including the following covariates: age, age^2^, sex, center, first 20 genotyping PCs. Only SNPs with MAF > 0.005 and imputation INFO score > 0.80 were included (UKBB, *n* = 223; UKBL, *n* = 190; both, *n* = 149). Results were then meta-analyzed using inverse variance weighting.

#### Identification of independent genetic effects

Single SNP study-wide significance was established by generating 300 normally distributed dummy traits, and running single SNP tests using the UKBB data. The minimum SNP *p* value for each dummy trait was then selected, and the 15th most significant *p* value from the 300 analyses was divided by 42 (41 real traits + mtDNA-CN), resulting in a study-wide *p* value threshold of *P* < 9.5 × 10^–6^. To identify a subset of traits to perform credible set identification using SusieR (see above, Fine Mapping), SNPs were first filtered based on the study-wide *p* value threshold, and then most significantly associated trait was identified for each SNP. SusieR, (parameters: *L* = 10, estimate_residual_variance = TRUE, estimate_prior_variance = TRUE, check_z = FALSE) was then run for each of these traits using the UKBB imputed data and summary association test statistics. A total of seven credible sets were identified across the four traits, two of which co-localized, resulting in six credible sets. Independence across the six credible sets was tested using multivariate regression models, and requiring *P* < 0.0005 for at least one trait for a SNP to remain in the model. SNPs MT73A_G and MT 7028C_T were in moderate-high LD (*r*^2^ = 0.67), but based on conditional regression analyses as described in the main results, capture independent effects and are associated with different traits.

#### Haplotype generation and analysis

Haplotype was constructed by concatenating SNPs across the six credible sets using SNPs directly genotyped on both genotyping arrays. This required selecting a SNP with a lower PPI for two of the six credible sets (MT12612A_G replaced MT462C_T, *r*^2^ = 0.81; MT10238T_C replaced MT4529A_T, *r*^2^ = 0.89). Haplotypes with MAF < 0.005 were set to missing (*n* = 1607), resulting in eight haplotypes, with the most common haplotype set as reference. Significance for haplotype associations with each trait was generated by an anova between regression models with and without the haplotypes. Covariates included age, age^2^, sex, center, first 40 genotyping PCs, and genotyping array.

Mortality analyses were run using Cox proportional hazards models, with covariates as above. Individuals with external causes of death (ICD 10 Death Code categories V, W, X, Y) were censored at the time of death. Additionally, for non-cancer mortality analyses, cancer death (ICD 10 Death Code categories C00-D48) was censored. For cancer mortality analyses, all death due to non-cancer cases were censored at the time of death.

Clustering for visualization was performed using the R package ‘heatmaply’, with default setting and hclust_method = ”ward.D2”.

All statistical analyses were performed using R version 4.0.3.

## Results

### Sample characteristics

The current study included 465,809 White individuals (53.9% female) with an average age of 56.6 yrs (sd = 8.2 yrs) (Supplemental Table 1). Follow-up validation analyses were performed in 4770 Black individuals (60.2% female) with an average age of 61.2 yrs (sd = 7.4 yrs). The majority of the data originated from the UKB (93%). The bulk of the DNA used for mtDNA-CN estimation was derived from the buffy coat (95.5%) while the rest was derived from peripheral leukocytes (2.2%), whole blood (2.3%), or brain (< 0.2%). mtDNA-CN estimated from Affymetrix genotyping arrays consisted of 97.9% of the data while the remainder was derived from qPCR (1.8%) and WGS (0.3%).

### GWAS reveals 97 loci that are significantly associated with mtDNA-CN

**Fig. 1:**
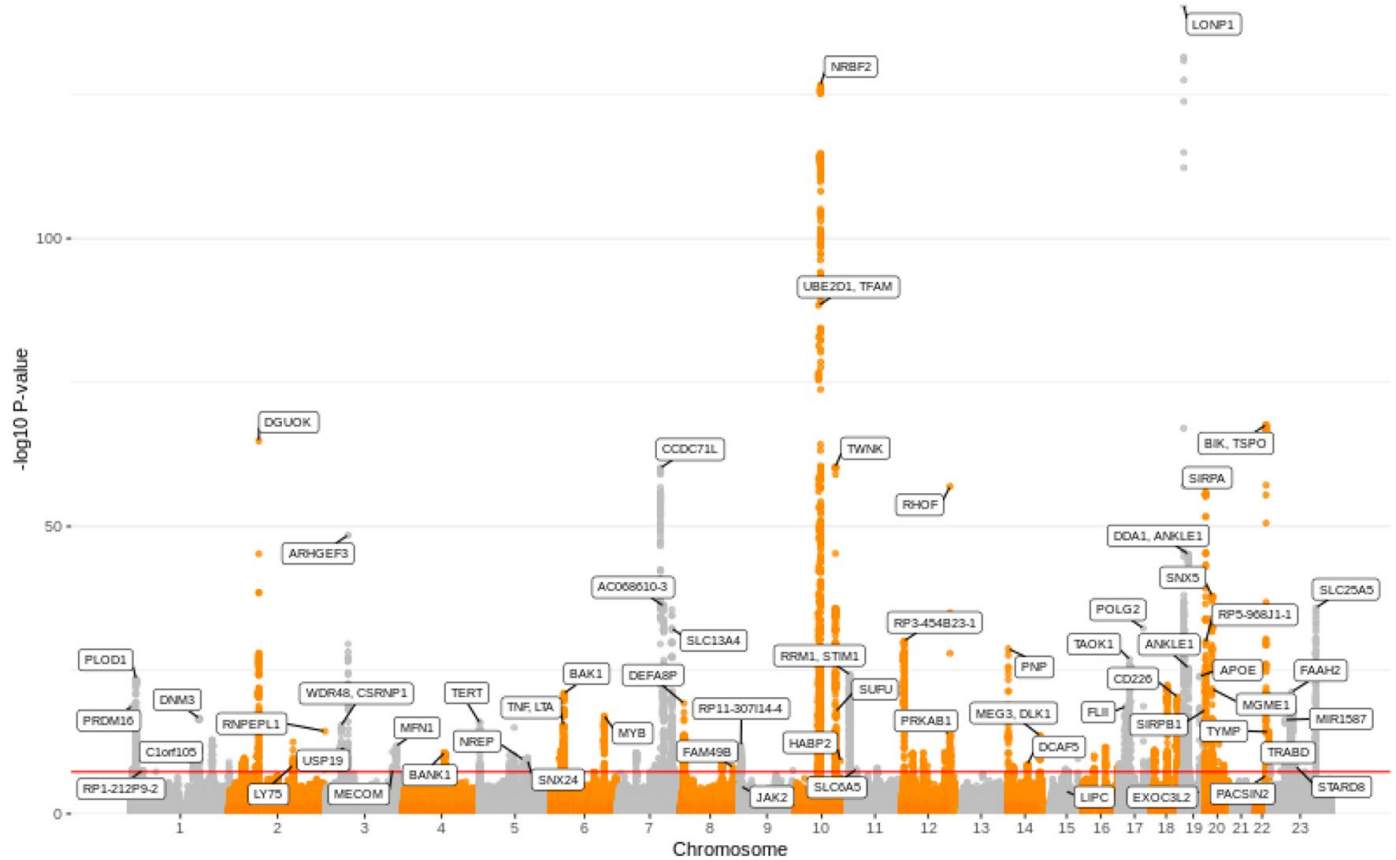
Manhattan plot of GWS loci from UKB-only analyses. Manhattan plot showing genome-wide significant loci for the UK Biobank-only analyses

Previous work has demonstrated that the method used to measure mtDNA-CN can impact the strength of association (Longchamps et al. 2020). To account for potential differences across studies due to the different mtDNA-CN measurements used, as well as the inclusion of blood cell counts as covariates in only a subset of the cohorts, we took two approaches. First, we used a random-effects model to perform meta-analyses, allowing for different genetic effect size estimates across cohorts. Second, we performed three complementary analyses in individuals who self-identified as White: (1) meta-analysis of all available studies (*n* = 465,809); (2) meta-analysis of studies with available data for cell count adjustment (*n* = 456,151); and (3) GWAS of UKB only, adjusting for age, sex, and cell counts (*n* = 440,266) (Fig. 1). 77 loci were significant in all three meta-analyses, and we identified 93 independent loci that were significant in at least one of the analyses. In the meta-analysis of UKB-only data, 92 of the total 93 loci were identified (Supplemental Fig. 4). Given that > 90% of the samples come from the UKB study, and the challenge of interpreting effect size estimates from a random-effects model, downstream analyses all use effect size estimates from the UKB only analyses (Supplemental Table 6), which showed no evidence for population substructure inflating test statistics, with a genomic inflation factor of 1.09 (Supplemental Fig. 5). mtDNA-CN in the UKB dataset was significantly associated with known covariates such as age (*p* < 2 × 10^–16^) and sex (*p* < 2 × 10^–16^) in the expected directions, with older individuals having lower mtDNA-CN, and females having higher mtDNA-CN. SNP heritability estimated from BOLT-LMM (Loh et al. 2015) for mtDNA-CN adjusted for age and sex was 10.5% while heritability for mtDNA-CN adjusted for age, sex, and cell counts was 7.4%, implying that some of the mtDNA-CN heritability observed in previous studies could be due to heritability of cell-type composition. We also used SumHer (Speed 2019) as an alternative approach to calculating SNP heritability, which returned a comparable estimate of 7.0% for the cell-count corrected mtDNA-CN metric.

The most significant SNP associated with mtDNA-CN was a missense mutation in *LONP1* (*p* = 3.00 × 10^–141^), a gene that encodes a mitochondrial protease that can directly bind mtDNA, and has been shown to regulate *TFAM*, a transcription factor involved in mtDNA replication and transcription (for review see Gibellini et al*.*) (Gibellini et al. 2020).

Meta-analysis of the sex-stratified X chromosome results identified four loci significantly associated with mtDNA-CN, with directionality consistent across the male and female stratified analyses (Supplemental Table 7). We note that there are similar results for the significant autosomal results, with effect sizes being highly concordant between sexes save for a single rare variant (MAF < 0.005), whose sex interaction *p* value does not pass multi-test correction (*p* = 0.003, Supplemental Fig. 6).

### Fine-mapping and secondary hits uncover multiple independent signals within loci

To identify additional independent SNPs within novel loci whose effects were masked by the original significant SNP, as well as identify additional loci, we took two approaches. First, a conditional analysis adjusting for the top 93 SNPs from the initial (primary) GWAS run revealed three novel loci and 19 additional independent significant SNPs within existing loci. We also performed fine-mapping with susieR (Wang et al. 2020) and discovered an additional 14 independent SNPs within existing loci. The majority of loci had only one 95% credible set of SNPs; further, twenty of the credible sets contained only one SNP. However, many of the credible sets contained greater than 50 SNPs after fine-mapping, and 12 of the 122 credible sets had a missense SNP as the SNP with the highest PIP in the set. Using these two methods, we identified in total 129 independent SNPs across 96 autosomal loci (Supplemental Fig. 7), while susieR fine-mapping and conditional analyses for the X-chromosome loci did not reveal any additional secondary signals.

### Replication of previously identified signals and discovery of additional GWS signals

Out of the 50 loci reported in Hägg et al. 2020 (Hägg et al. 2020), we replicate 38 loci in our cell-count adjusted analyses (Supplemental Table 8). As the two GWASs both use UK Biobank data, this replication is unsurprising. Out of the 12 loci that were not genome-wide significant in our cell-count adjusted analyses, 11 were significant when we did not adjust our mtDNA-CN metric for cell counts, suggesting that cell-type composition may be driving these signals. The current manuscript also reports 62 additional loci that are not in the Hägg et al. 2020 study. This is likely due to increased power, as the sample size used for the current analyses is nearly twice as large.

### Associations in Black populations show concordance between nuclear genetic effects

**Fig. 2:**
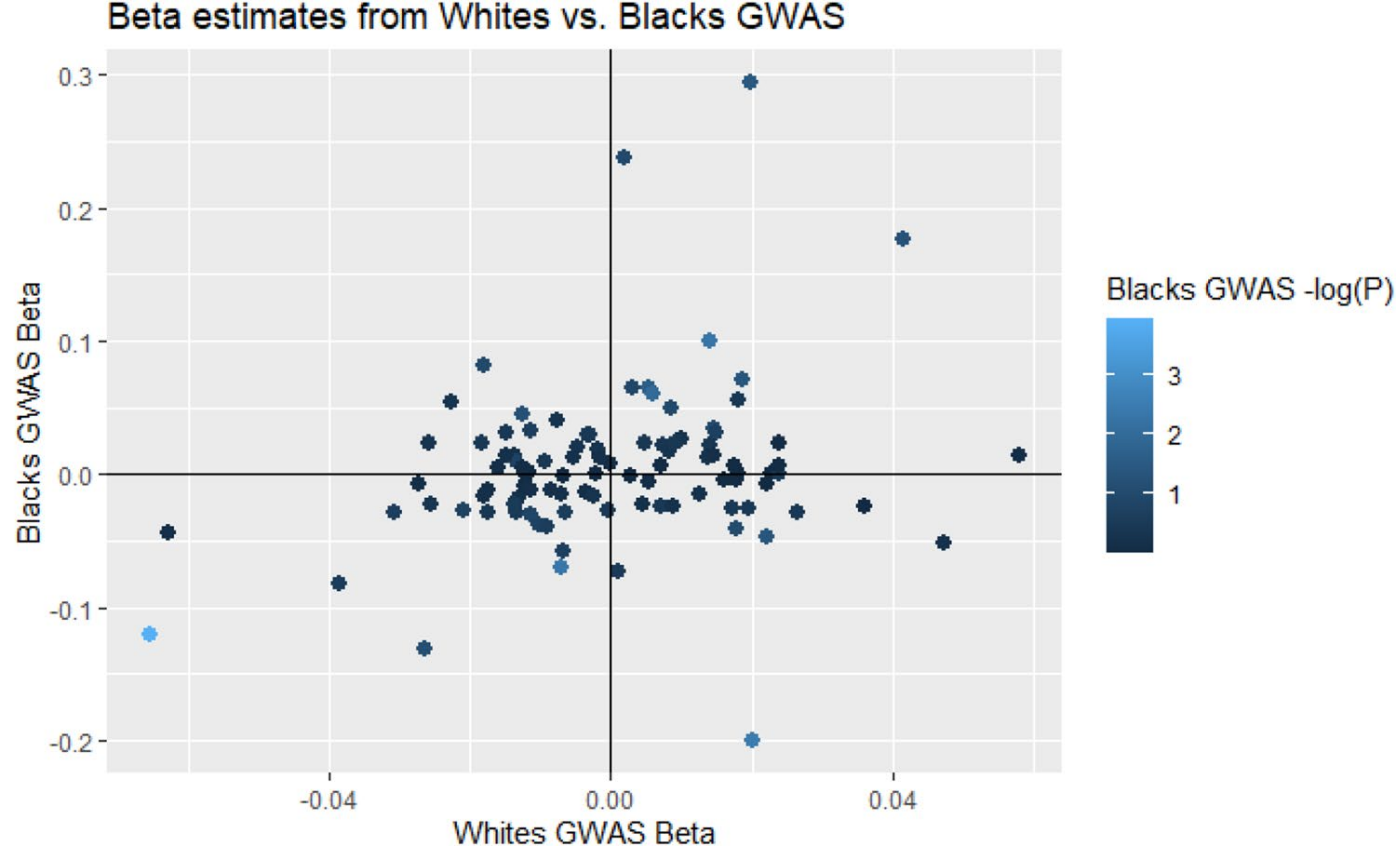
Scatterplot displaying effect size estimates between White/Black individuals GWAS results for the 129 autosomal SNPs identified in the Whites analyses. Scatterplot showing a comparison between effect size estimates for White and Black individuals. Color represents significance of effect for each locus in Black individuals GWAS analyses

Examining the 129 autosomal SNPs from the Whites-only analysis, 99 were available in the Black individuals-only meta-analysis (*n* = 4770). After multiple testing corrections, one of these SNPs was significant (rs73349121, *p* = 0.0001), 9 were nominally significant (*p* < 0.05, with 5 expected), and 58/99 had a direction of effect that was consistent with the Whites-only analyses (one-sided *p* = 0.04, Fig. 2). Despite being under-powered, these results in the Black individuals-only analyses provide evidence for similar nuclear genetic effects in a different ancestry group. As African mtDNA haplogroups are substantially different from those of Europeans, we additionally examined associations between haplogroups and mtDNA-CN. While none of the associations achieve genome-wide significance, 3/10 haplogroups are significant at *P* < 0.05, and 2 are significant after Bonferroni correction for the number of haplogroups tested (Supplemental Table 9).

### Significant enrichment of mtDNA depletion syndrome genes after gene prioritization

We integrated results from three different gene prioritization and functional annotation methods (ANNOVAR (Wang et al. 2010), COLOC (Giambartolomei et al. 2014), and DEPICT (Pers et al. 2015)) so that loci with nonsynonymous variants in gene exons were prioritized first, with eQTL co-localization results considered second (Supplemental Table 10), and those from DEPICT (Supplemental Table 11) were considered last (Supplemental Fig. 3). For 20 loci, multiple genes were assigned as analyses could not identify a single priority gene (Supplemental Table 12). As eQTLGen did not evaluate X chromosome variants and none of the credible sets contained missense variants, the four X-chromosome loci were assigned to the nearest gene.

We noted the identification of a number of mtDNA depletion syndrome genes in the priority list and tested for enrichment of these known causal genes using a one-sided Fisher’s exact test. For this analysis, all genes for loci assigned to multiple genes were used, and genes for all primary and secondary loci were considered. Our gene prioritization approach identified seven of 16 mtDNA depletion genes (Supplemental Table 13), consistent with a highly significant enrichment (one-sided *p* = 3.09 × 10^–15^).

### Gene set enrichment analyses show significant enrichment of mitochondria-related terms

To avoid bias from a single locus with multiple functionally related genes contributing to a false-positive signal, only one gene per unique locus was used, prioritizing genes assigned to primary loci. For loci with multiple assigned genes, one gene was randomly selected for testing. To test for the robustness of gene set enrichment results, random selection was repeated 50 times, and only gene sets that were significantly enriched for at least 40 iterations were retained. In all, a total of 100 genes were utilized for GO term and KEGG pathway enrichment analyses. Using a Bonferroni-corrected p-value cutoff, 12 gene sets were significantly enriched for all 50 iterations, including mitochondrial nucleoid, mitochondrial DNA replication, and amyloid-beta clearance (Supplemental Table 14). No KEGG terms were significant across multiple iterations.

### Predicted gene expression for multiple mitochondrial genes is associated with GWS mtDNA-CN SNPs

**Fig. 3:**
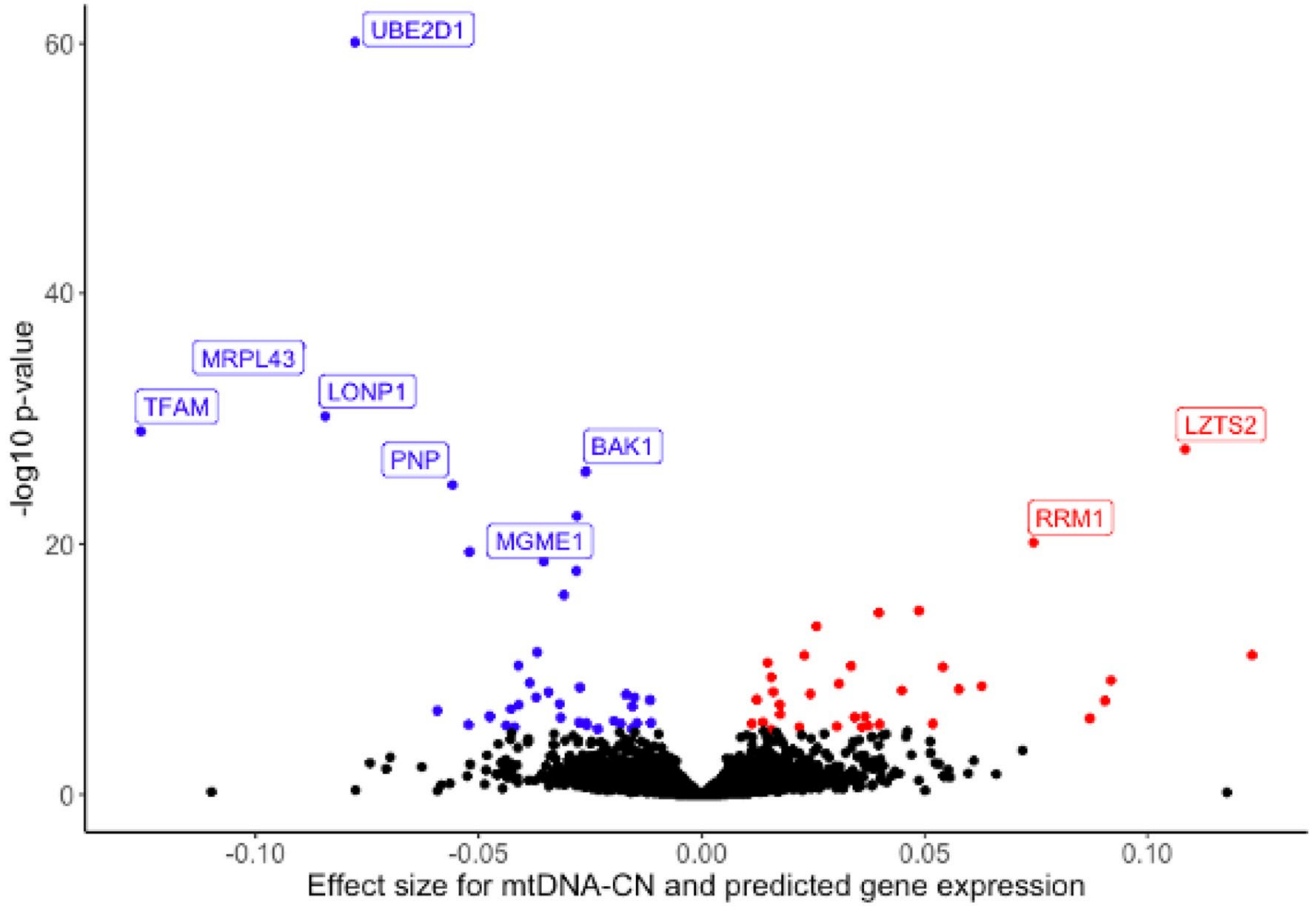
Volcano plot of genes whose predicted gene expression is significantly associated with mtDNA-CN. Volcano plot showing genes whose predicted gene expression is significantly associated with mtDNA-CN. Red indicates positive associations, blue indicates negative associations. Three genes (ARRDC1, EHMT1, PNPLA7) had extreme effect size estimates greater than 0.3 but were non-significant and removed from the plot for readability

As a complementary approach to single-SNP analyses, we explored the associations between mtDNA-CN and predicted gene expression using MetaXcan (Barbeira et al. 2018). MetaXcan incorporates multiple SNPs within a locus along with a reference eQTL dataset to generate predicted gene expression levels. As our study estimated mtDNA-CN derived from blood, we used whole blood gene expression eQTLs from the Gene-Tissue Expression (GTEx) consortium (GTEx Consortium 2013) to predict gene expression in the UKB dataset. We identified 6285 genes that had a predicted performance p-value of less than 0.05 (i.e., they had sufficient data to generate robust gene expression levels) and were tested for association with mtDNA-CN. Of these genes, 74 were significantly associated with mtDNA-CN (*p* < 7.95 × 10^–6^) (Fig. 3, Supplemental Table 15), including eight that were not identified through single-SNP analyses. Many of the significant genes have known mitochondrial functions, notably the mtDNA transcription factor *TFAM* (*p* = 1.09 × 10^–29^) and mitochondrial exonuclease *MGME1* (*p* = 5.87 × 10^–23^), genes known as causal for mtDNA depletion syndromes (Stiles et al. 2016; Kornblum et al. 2013). Additionally, *LONP1*, *MRPL43*, and *BAK1*, are all genes with known mitochondrial functions (Liu et al. 2004; Sharma et al. 2003; Shimizu et al. 1999). Bonferroni significant MetaXcan genes were used for gene enrichment analysis, finding enrichment for “nucleobase metabolic process” (*p* = 1.47 × 10^–4^) and “mitochondrial fusion” (*p* = 1.86 × 10^–4^) (Supplemental Fig. 7).

### PHEWAS-based SNP clustering and gene set enrichment uncover 3 sets of SNPs mapping to distinct biological pathways

**Fig. 4:**
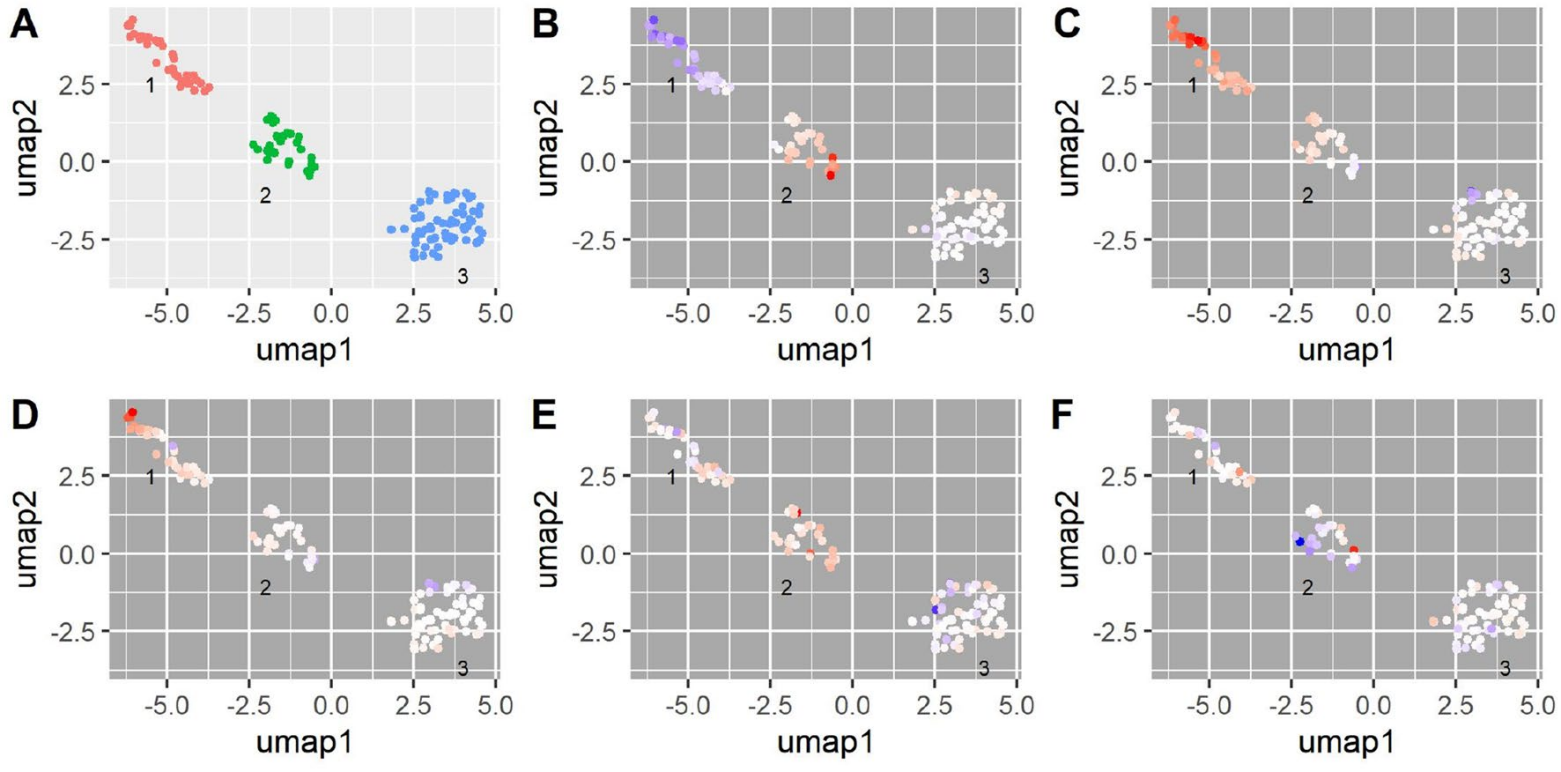
PHEWAS-based clustering of mtDNA-CN associated SNPs. UMAP clusters created from PHEWAS associations for mtDNA-CN associated SNPs. **A** Three clusters were identified as labeled in the panel; orange indicates no cluster. **B**–**F** SNPs are colored based on their effect estimate size, standardized to the effect on mtDNA-CN (red = positive, blue = negative estimates), for **B** platelet count, **C** mean platelet volume, **D** platelet distribution width, **E** serum calcium levels, **F** mean spherical cellular volume

mtDNA-CN is associated with numerous quantitative and qualitative phenotypes, many of which are relevant to aging-related disease (Dai et al. 2012; Cui et al. 2012; Herst et al. 2017; Ashar et al. 2015, 2017; Tin et al. 2016; Pyle et al. 2016; Wei et al. 2017; Reznik et al. 2016). We hypothesized that this pleiotropy may reflect different underlying functional domains captured by mtDNA-CN, and may be reflected in GWAS-identified SNPs and their likely causal genes. To test this hypothesis, we used the UKB data to identify quantitative traits associated with mtDNA-CN and selected 41 highly significant, non-redundant traits to test for association with the mtDNA-CN GWAS SNPs (Supplemental Table 5, in PHEWAS = 1). We clustered SNPs using the trait effect size (beta) divided by the mtDNA-CN effect size estimate so that all effects are standardized to the effect of the mtDNA-CN increasing allele for each locus. We identified 3 clusters of SNPs (Supplemental Table 16, Fig. 4A), with cluster 1 containing SNPs in which the mtDNA-CN increasing allele is associated with decreased platelet count (PLT) (Fig. 4B), increased mean platelet volume (MPV) (Fig. 4C), and platelet distribution width (PDW) (Fig. 4D), consistent with a role in platelet activation (Vagdatli et al. 2010). Cluster 2 is most strongly enriched for SNPs in which the mtDNA-CN increasing allele is associated with increased PLT, plateletcrit (PCT, a measure of total platelet mass), serum calcium (Fig. 4E), serum phosphate, as well as decreased mean corpuscular volume (MCV) and mean spherical cellular volume (Fig. 4F) (Supplemental Table 17). The cluster 2 phenotypes implicate megakaryocyte proliferation and proplatelet formation in addition to apoptosis and autophagy, and are supported by the genes identified for this cluster (megakaryocyte proliferation and proplatelet formation: *MYB*, *JAK2* (PathCards : Factors involved in megakaryocyte development and platelet production Pathway and related pathways. xxxx), apoptosis and autophagy: *BAK1*, *BCL2, TYMP*) (PathCards : Apoptosis and Autophagy Pathway and related pathways. xxxx). Gene set enrichment analysis confirmed this, as cluster 2 genes are significantly enriched for extrinsic apoptosis signaling pathways in the absence of ligand (Supplemental Table 18). Cluster 3 did not yield any specific trait enrichment (all significant results reflected the strong enrichment observed in clusters 1–2); however, gene set enrichment for this cluster identified multiple mtDNA-related gene ontology terms, including mitochondrial DNA replication, gamma DNA polymerase complex, and mitochondrial nucleoid (Supplemental Table 19).

### Determination of causal associations between mitochondrial function and mtDNA-CN associated traits using mitochondrial SNPs

**Fig. 5:**
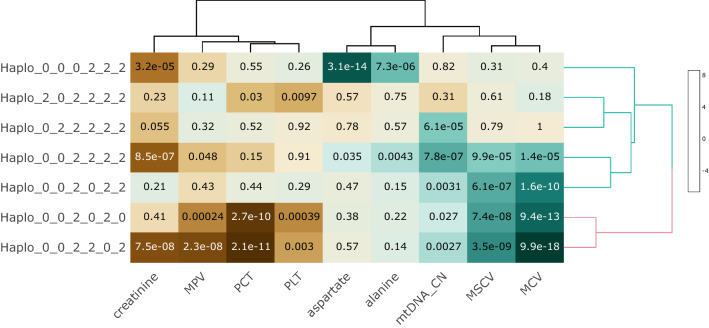
Associations between mtDNA-CN associated phenotypes and mitochondrial haplotypes. Mitochondrial haplotypes are significantly associated with mtDNA-CN associated traits, implying causal relationships between mitochondrial function and traits of interest. Haplotypes are notated in the following format: MT73_MT7028_MT10238_ MT12612_MT13617_MT15257

The extensive pleiotropy and limited variance explained of nuclear DNA SNPs associated with mtDNA-CN (< 1% of the variance in mtDNA-CN explained by GWS loci when predicted into the ARIC cohort) precludes the use of traditional Mendelian randomization (MR) approaches to establish causality between mtDNA-CN and the 41 identified mtDNA-CN associated traits. As an alternative approach, we examined the association of mitochondrial SNPs with mtDNA-CN and the 41 traits, under the assumption that these SNPs can only act through alteration of mitochondrial function, and thus a significant association implies causality. Imputation and analyses of mitochondrial SNPs were run stratified by genotyping array (see Methods), and then meta-analyzed using inverse-variance weighting. After multi-test correction (*P* < 9.5 × 10^–6^), we identified 45 SNPs associated with one or more of the traits, ranging from 1 to 6 traits per SNP. To identify independent effects, we first identified the most significantly associated trait for each SNP, highlighting four traits (aspartate aminotransferase, creatinine, MCV, PCT) in which to run susieR to identify independent credible sets. We identified six independent effects across the four traits, with MCV credible set 4 and platelet credible set 1 representing the same effect. We note that two of the SNPs are in moderately high LD (MT73A_G and MT7028C_T, *r*^2^ = 0.67), however, conditional analyses demonstrate that MT73A_G is associated with creatinine, and not MCV, and the reverse is seen for MT7028C_T (Supplemental Table 20). Leveraging the haploid nature of the mitochondrial genome, we selected the directly genotyped SNP with the highest PIP from each credible set (Supplemental Table 21), and identified eight haplotypes with MAF > 0.005 (Supplemental Table 22). As haplotypes indicate regions of the mitochondrial genome that are in linkage disequilibrium, some of them may be captured by ancestral haplogroups (Supplemental Table 23). For example, nearly all the individuals in haplotype 1 belong to haplogroup N1. As a direct test of whether haplogroup explains the observed association, we included haplogroup as a covariate in the regression model for MCV, and demonstrate that while somewhat attenuated, haplotype is still highly significant (*P* < 1.3 × 10–10 vs. *P* < 7.2 × 10–27). Comparing linear regression models with and without the haplotypes in the model, we identify 14 traits nominally associated (*p* < 0.05), and nine traits significantly associated after Bonferroni correction, with mtDNA genetic variation (Supplemental Table 24, Fig. 5). These results causally implicate mitochondrial function in a variety of cell-related traits (MCV, MSCV, MPV, PCT, Platelet), kidney function (creatinine), liver function (aspartate and alanine aminotransferases) and mtDNA-CN.

### Association of mitochondrial haplotypes with mortality

We have previously shown that mtDNA-CN is associated with overall mortality (Ashar et al. 2015). As above, we collectively tested the mitochondrial haplotypes for association with mortality not due to external causes (e.g., no accidents, falls, see Methods; *n* = 24,622, median follow-up time = 4318 days), and found a nominally significant association with overall mortality (*p* = 0.044, Supplemental Table 25). Given the conflicting reports between increased mtDNA-CN and both increased and decreased cancer risk (Reznik et al. 2016; Yuan et al. 2020; Mizumachi et al. 2008), we looked separately at cancer (*n* = 13,231) and non-cancer mortality (*n* = 11,391). While there was no association between mitochondrial haplotypes and cancer mortality (*p* = 0.74), we saw a highly significant association with non-cancer mortality (*p* = 6.56 × 10^–4^).

## Discussion

We conducted a GWAS for mtDNA-CN using 465,809 individuals from the CHARGE consortium and the UKB. We report 133 independent signals originating from 100 loci, the majority of which were not identified in previous studies. Examining our GWS SNPs in a Black population, we observed a concordant signal, suggesting that the nuclear genetic etiology of mtDNA-CN may be broadly similar across populations. Using several functional follow-up methods, genes were assigned for each identified independent hit and significant enrichment was observed for genes involved in mitochondrial DNA metabolism, homeostasis, cell activation, and amyloid-beta clearance. In total, we assigned 128 unique genes to independent GWAS signals associated with mtDNA-CN. We also identified eight additional genes whose predicted gene expression is associated with mtDNA-CN that could not be mapped back to GWS loci. Finally, using a clustering approach based on SNP associations with various mtDNA-CN associated phenotypes, we were able to functionally categorize SNPs, providing insight into biological pathways that impact mtDNA-CN.

We note that during the preparation of this manuscript, a GWAS for mtDNA-CN performed in 295,150 unrelated individuals from the UK Biobank was published, which reported 50 genome-wide significant regions (Hägg et al. 2020). Within our GWAS, we replicate 38 of these 50 genome-wide significant loci in our cell count corrected analyses. An additional 11 out of the remaining 12 loci are genome-wide significant when we do not adjust mtDNA-CN for cell count. While Hagg et al adjust for cell-type composition, this difference suggests that their adjustment may not be fully capturing the effects of cell counts. Additionally, our analyses report 59 additional loci that are not observed in the previous paper, largely due to the increased power of our study.

We were able to identify a substantial proportion of the genes involved in mtDNA depletion syndromes (7/16, *p* = 3.09 × 10^–15^ for enrichment), including *TWNK*, *TFAM*, *DGUOK*, *MGME1*, *RRM2B, TYMP,* and *POLG*. mtDNA depletion syndromes can be broken down into 5 subtypes based on their constellation of phenotypes (Basel 2020), and with the exception of cardiomyopathic subtypes (associated with mutations in *AGK* and *SLC25A4*), we were able to identify at least one gene from the other four subtypes, suggesting that our mtDNA-CN measurement in blood-derived DNA can identify genes widely relevant to non-blood phenotypes. This finding is consistent with a large body of work showing that mtDNA-CN measured in blood is associated with numerous aging-related phenotypes for which the primary tissue of interest is not blood (e.g. chronic kidney disease (Tin et al. 2016), heart failure (Hong et al. 2020), and diabetes (DeBarmore et al. 2020)). Also consistent with this finding is recent work demonstrating that mtDNA-CN measured in blood is associated with mtRNA expression across numerous non-blood tissues, suggesting a link between mitochondrial activity measured in blood and other tissues (Yang et al. 2021). However, vascular dysfunction is common to these different diseases, and it is important to note that mtDNA-CN in blood could have a direct effect on disease etiology, rather than simply serving as a reflection of mtDNA-CN in primary disease tissues.

In addition to identifying the mtDNA depletion syndrome genes directly linked to mitochondrial DNA metabolic processes, DNA replication, and genome maintenance, we also identify genes which play a role in mitochondrial function. The top GWAS hit is a missense mutation in *LONP1*, which encodes a mitochondrial protease that has been shown to cause mitochondrial cytopathy and reduced respiratory chain activity (Hannah-Shmouni et al. 2019; Grainha et al. 2018). Interestingly, this missense mutation was recently found to be associated with mitochondrial tRNA methylation levels (Ali et al. 2020). Additional genes known to impact mitochondrial function include *MFN1*, which encodes a mediator of mitochondrial fusion (Schrepfer and Scorrano 2016; Ishihara et al. 2004), *STMP1*, which plays a role in mitochondrial respiration (Zhang et al. 2012), and *MRPS35*, which encodes a ribosomal protein involved in protein synthesis in the mitochondrion (Cavdar Koc et al. 2001; Márquez-Jurado et al. 2018).

Using a combination of gene-based tests and gene prioritization using functional annotation we assigned genes to identified signals. However, a caveat is that these computational predictions must be functionally validated in a biological system to definitively assign underlying responsible genes. After assigning genes using the specified criteria, pathway analyses reveal enrichment for numerous mitochondrial related pathways, as well as those involved in the regulation of cell differentiation (*p* < 1.08 × 10^–5^), homeostatic processes (*p* < 3.77 × 10^–6^), and cellular response to stress (*p* < 3.49 × 10^–6^) (Supplemental Table 13). These results provide additional evidence for the broad role played by mitochondria in numerous aspects of cellular function. Of particular interest, the GO term for amyloid beta is significantly enriched, reinforcing a link between mtDNA-CN and neurodegenerative disease (Dölle et al. 2016; Chen et al. 2019; Pinto and Moraes 2014). Previous work from our lab using the UKB has shown that higher mtDNA-CN is associated with lower rates of prevalent neurodegenerative disease, and is predictive of decreased risk of incident neurodegenerative disease (Yang et al. 2021). mtDNA-CN is also known to be decreased in the frontal cortex of Alzheimer’s disease (AD) patients (Rodríguez-Santiago et al. 2001). Interestingly, the four GWAS-identified genes driving the enrichment for amyloid-beta clearance are all related to the regulation of lipid levels, and lipid homeostasis within the brain is known to play an important role in Alzheimer’s disease (Chew et al. 2020). *APOE*, one of the most well-known risk genes for Alzheimer’s disease, is a cholesterol carrier involved in lipid transport, and the ApoE-ɛ4 isoform involved in AD pathogenesis is associated with mitochondrial dysfunction and oxidative distress in the human brain (Yin et al. 2020); *CD36* is a platelet glycoprotein which mediates the response to amyloid-beta accumulation (Khoury et al. 2003); *LDLR* is a low-density lipoprotein receptor associated with AD (Lämsä et al. 2008); and *ABCA7* is a phospholipid transporter (Tomioka et al. 2017). *ABCA7* loss of function variants are enriched in both AD and Parkinson’s disease (PD) patients (Nuytemans et al. 2016), suggesting a broad role across neurodegenerative diseases.

Given the integral role of mitochondria in cellular function, from ATP formation and energy production, signaling through reactive oxygen species, and apoptosis mediation, there is a strong basis to a priori assume that genetic variants associated with mtDNA-CN are likely to be highly pleiotropic. It has also been shown that mtDNA variants themselves are pleiotropic and are capable of affecting oxidative phosphorylation and gene expression (Cohen et al. 2016; Gómez-Durán et al. 2010; Marom et al. 2017). MtDNA-CN itself is associated with numerous phenotypes, suggesting that multiple biological pathways are involved in mtDNA-CN control (Supplemental Table 5). Through our PHEWAS-based clustering approach using 41 mtDNA-CN associated phenotypes, we uncovered phenotypic associations between three distinct clusters of GWS mtDNA-CN associated SNPs. Cluster 1 was characterized by increased MPV, PDW, and decreased PLT (note that measured MPV and PLT are generally inversely correlated to maintain hemostasis), which are the hallmarks of platelet activation (Vagdatli et al. 2010). The link between platelets and mtDNA-CN has typically revolved around platelet count, as platelets have functional mitochondria, but do not have a nucleus. Given that the mtDNA-CN measurement is the ratio between mtDNA and nuclear DNA, increased platelets, all else being equal, would directly equate with increased mtDNA-CN. We note that the mtDNA-CN metric used in this GWAS was adjusted for platelet count, likely increasing the ability to detect variants that impact mtDNA-CN through increased platelet activation. Examining the genes within this cluster suggests roles for actin formation and regulation (*TPM4*, *PACSIN2*) (Crabos et al. 1991; Kostan et al. 2014) and vesicular transport and endocytic trafficking (*DNM3*, *EHD3*) (Sever 2002; Cai et al. 2013) in platelet activation.

Cluster 2 is most strongly enriched for SNPs in which the mtDNA-CN increasing allele is associated with increased PLT/PCT and serum calcium/phosphate. Examining the genes assigned to the cluster, we implicate megakaryocyte proliferation and proplatelet formation (*MYB*, *JAK2*) (PathCards : Factors involved in megakaryocyte development and platelet production Pathway and related pathways. xxxx), and apoptosis and autophagy (*BAK1*, *BCL2, TYMP*) (PathCards : Apoptosis and Autophagy Pathway and related pathways. xxxx). Megakaryocytes are used to form proplatelets, and the process includes an important role for both intra- and extracellular calcium levels (Buduo et al. 2014). A role for apoptosis, and specifically *BCL2*, in proplatelet formation and platelet release has been suggested (Botton et al. 2002; Josefsson et al. 2011), however, work in mice has suggested that apoptosis does not play a direct role in these processes (Josefsson et al. 2014). Nevertheless, apoptosis is important for platelet lifespan (McArthur et al. 2018).

Cluster 3 was particularly challenging to interpret, given that no particular phenotype was enriched relative to the non-cluster 3 SNPs. We note that this cluster appeared to be enriched for the mtDNA depletion syndrome genes, containing 6/7 genes identified in the GWAS, and significantly enriched for GO Terms related mitochondrial DNA. Additionally, genes in cluster 3 were significantly enriched for low-density lipoprotein particle binding, suggesting a role for lipid homeostasis. Closer inspection of cluster 3 genes reveals a number of genes known to be associated with lipid levels (*LIPC*, *CETP*, *LDLR*, *APOE*). While lipids play a role in both energy metabolism (largely through fatty acids) and cellular membrane formation, a link to mtDNA-CN and/or mitochondrial function is not well-established. One potentially interesting result is provided by Olkowicz and colleagues, who demonstrated that ApoE^−/−^/LDLR^−/−^ mice had increased cardiac mitochondrial oxidative metabolism, with proteomic analysis suggesting increased mitochondrial abundance in mouse hearts (Olkowicz et al. 2021). However, we note that our results show an association between decreased lipids and increased mtDNA-CN, rather than the reverse, shown in the Olkowicz study.

A strong rationale for the study of mtDNA-CN is the underlying assumption that it reflects mitochondrial function and is readily measured, often from existing data. A serious complication to the interpretation of the role of mitochondrial function in various traits has been use of blood-derived measurements, which can be confounded by differences in cell counts across individuals. Mendelian randomization has been widely used to infer causality between traits (e.g. LDL and CAD) (Linsel-Nitschke et al. 2008), but is only robust under conditions of little to no pleiotropy (Lawlor et al. 2008) and its power is a function of variance explained. For mtDNA-CN, the extensive pleiotropy and small amount of variance explained of GWS variants (< 1%) prevents the use of traditional MR approaches. As an alternative approach, we analyzed associations between mitochondrial DNA variants and mtDNA-CN-associated phenotypes. Presumably, variants located on the mitochondrial genome are only able to modify phenotypes through modulating mitochondrial function, allowing for causal inference. Our analyses revealed significant relationships between mitochondrial variants and creatinine, aspartate aminotransferase, MCV, and PCT. Creatinine and aspartate aminotransferase are markers of kidney and liver function respectively, and supporting these findings, mtDNA-CN has been linked to both chronic kidney disease (Tin et al. 2016) and non-alcoholic fatty liver disease (Sookoian et al. 2010). We also find a highly significant association between mitochondrial variation and non-cancer mortality, adding evidence for a causal relationship to previous findings showing mtDNA-CN is associated with all-cause mortality (Ashar et al. 2015).

Several limitations should be noted. First, despite the large sample size and numerous loci identified, we are likely missing a great deal of the true signal, as our SNP heritability estimates through SumHer and BOLT-LMM were 7.0% and 7.4% respectively, while previous studies have estimated mtDNA-CN heritability to be 65% (Xing et al. 2008). Second, while we have adjusted our mtDNA-CN metric for a variety of confounders, it is important to note that mtDNA-CN can be influenced by a variety of environmental factors including smoking (Vyas et al. 2020) and drugs, which have not been adjusted for in these analyses. Moreover, mtDNA-CN is not a direct reflection of mitochondrial function, which can confound interpretation. Finally, for analyses involving mitochondrial SNPs, since much of the mitochondrial genome is in LD, the selected mitochondrial SNPs may be in LD with the true causal SNP.

In summary, we performed the largest-to-date GWAS for mtDNA-CN, including almost 500,000 individuals. We identified three distinct groups of SNPs associated with mtDNA-CN that are related to platelet activation, megakaryocyte formation and apoptotic processes, and showed clear enrichment for genes involved in mtDNA depletion and nucleotide regulation. Additionally, we find that mitochondrial variants are significantly associated with creatinine, aspartate aminotransferase, MCV, and PCT, implying a causal relationship between mitochondrial function and these phenotypes. Finally, we provide strong evidence that mitochondrial function is causal for non-cancer mortality. Given the role of mtDNA-CN, and, by proxy, mitochondrial function in aging-related disease, this work begins to unravel the many varied underlying mechanisms through which mitochondrial function impacts human health.

## Supplementary Information

Below is the link to the electronic supplementary material.

Supplementary file1 (XLSX 24 KB)

Supplementary file2 (DOCX 571 KB)

Supplementary file3 (XLSX 476 KB)

## Data Availability

All data used in this manuscript is available through either the UKBiobank and CHARGE consortiums.

## ﻿Code availability

Code and scripts are available in a zipped file at https://www.arkinglab.org/resources/.
